# Scratching beyond the surface — minimal actin assemblies as tools to elucidate mechanical reinforcement and shape change

**DOI:** 10.1042/ETLS20220052

**Published:** 2022-12-21

**Authors:** Anders Aufderhorst-Roberts, Margarita Staykova

**Affiliations:** Centre for Materials Physics, Department of Physics, Durham University, Durham DH1 3LE, U.K.

**Keywords:** actin, bottom-up reconstitution, cell mechanics, membrane, myosin, shape change

## Abstract

The interaction between the actin cytoskeleton and the plasma membrane in eukaryotic cells is integral to a large number of functions such as shape change, mechanical reinforcement and contraction. These phenomena are driven by the architectural regulation of a thin actin network, directly beneath the membrane through interactions with a variety of binding proteins, membrane anchoring proteins and molecular motors. An increasingly common approach to understanding the mechanisms that drive these processes is to build model systems from reconstituted lipids, actin filaments and associated actin-binding proteins. Here we review recent progress in this field, with a particular emphasis on how the actin cytoskeleton provides mechanical reinforcement, drives shape change and induces contraction. Finally, we discuss potential future developments in the field, which would allow the extension of these techniques to more complex cellular processes.

## Introduction

**Figure 1.**
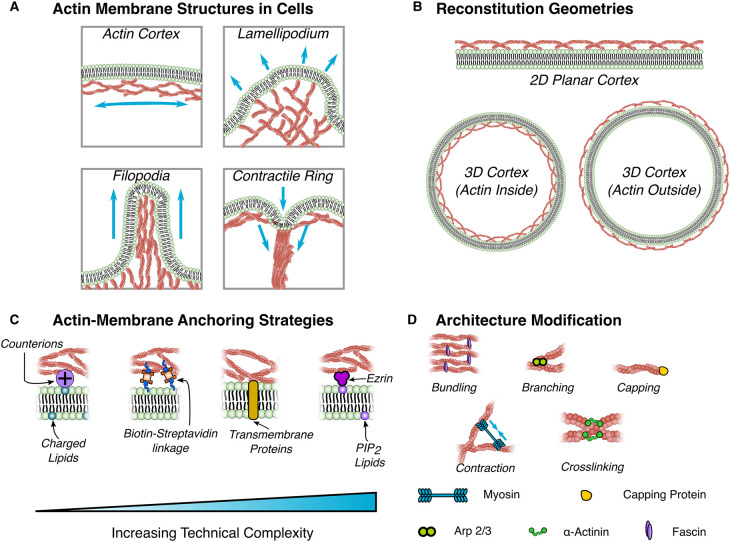
Key considerations in constructing reconsituted actin-membrane assemblies. (**a**) Actin networks in cells interact with the plasma membrane to form a variety of different structures. (**b**) Recreating these structures in reconstituted systems can be achieved through the assembly of actin filaments on a planar lipid bilayer or on the outside or inside leaflet of a giant unilamellar vesicle (GUV). (**c**) A key design consideration is the anchoring of the actin network to the membrane which can be achieved through a range of strategies, in order of approximate physiological accuracy: electrostatic interactions, the ligand receptor pair biotin-streptavidin, reconstituted transmembrane proteins and the anchoring protein ezrin. (**d**) Changes in actin network architecture can be achieved through a range of actin-binding proteins, the most commonly used of which are: the cross-linker *α*-actinin, the branching complex arp2/3, various capping proteins, the bundling protein fascin and motor proteins of the myosin-II family which induce contraction through sliding actin filaments past one another.

From a material perspective, cells are fascinating mechanical entities. Not only can they withstand the often-substantial external forces from their environment, but they can also generate the complex internal forces required to remodel, change shape and undergo motility. Mechanical disruption to these essential processes is invariably correlated with numerous cellular pathologies [1]. One integral subsystem of almost all animal cells is the actin cortex, which comprises of a 200 nm thick [2] meshwork of filamentous actin organized and bound to the plasma membrane through an assortment of associated proteins. A fundamental role of the cortex is to provide cells with rigidity and to mechanically reinforce the cell membrane surface [3]. In addition, the active remodelling of actin filaments into different assemblies (Figure 1a) exert net forces on the cell surface, allowing cells to change shape, move, uptake nutrients and divide. For example, the ability of the actin filaments to polymerize at one end and depolymerize at the other [4] results in the formation of membrane lamellipodia and filopodial protrusions at the leading edge of crawling cells [4,5,6]. Myosin II motor proteins bind to pairs of actin filaments causing them to slide past one another [7,8], which regulates actin network stiffness and induce contraction.

To understand the physical principles that determine these diverse processes, an increasingly utilized approach is to construct model systems from reconstituted lipids, actin and other carefully chosen proteins. In this mini-review, we provide a brief overview of the strategies by which membrane-bound actin assemblies can be re-constructed and outline how these approaches have been used to elucidate the processes of mechanical reinforcement, shape change and contraction. Finally, we give our perspective on the likely next steps by which these approaches can be extended to address the complexity of biomechanical processes in cells.

## The reconstituted actin-membrane assembly toolkit

Reconstituted actin assemblies on membranes involve the careful reduction in the cell to only the components that are strictly necessary to recreate and understand the biomechanical processes of interest (Figure 1a). The co-assembly of the two main components, i.e. actin network onto a lipid membrane (Figure 1b), can be achieved on a planar-supported lipid bilayer [9] or on the surface of freestanding giant unilamellar vesicle (GUV), either on the outer leaflet [10] or on the inner leaflet [11]. The latter is generally seen to most closely resemble the native cell environment [9] but is more technically challenging and less amenable to external perturbation, since the actin mesh is encapsulated and therefore no longer accessible experimentally. The choice of the components that link the actin filaments onto the membrane (Figure 1c) or to each other (Figure 1d) depends on whether the study aims to understand the activity of a specific cellular component, or whether it seeks to reproduce the mechanism of a certain cellular process, in which case the components may not be those necessarily used by cells.

For example, to simply link an actin network to the membrane, electrostatic interactions [12], the ligand receptor pair biotin-streptavidin [5] or reconstituted transmembrane proteins [13,14] have been employed (Figure 1c). Other studies have specifically aimed to reconstitute ezrin — one of the main membrane-actin anchors in cells [15], in order to understand the conformational changes needed for its activation [16] and how its activity depends on the membrane curvature and composition [17,18]. Similarly, different strategies have been used to reconstitute the various actin architectures and functionalities observed in cells (Figure 1d). Cross-linking of actin networks has been achieved either by using the non-physiological biotin-streptavidin bond as a cross-linker [19] or *α*-actinin, which modifies network geometry and stiffness in cells [20]. Fascin has been reconstituted to study actin bundling observed in cell fillopodia [5,21], while the protein complex known as arp2/3 that branches actin filaments in the presence of a protein domain known as VCA, is used to form dendritic actin networks, found usually in cell lamellipodia [22,23,24]. In addition, model systems can also be used to understand how cells use auxiliary proteins such as cofilin, formin and capping proteins to regulate temporally and spatially their actin assemblies [24,23,22]. In the following, we will discuss how such systems have provided insights into the assembly and functionalities of actin cortices in cells and on the subtleties of their interactions with the lipid membrane.

## Mechanical reinforcement

**Figure 2.**
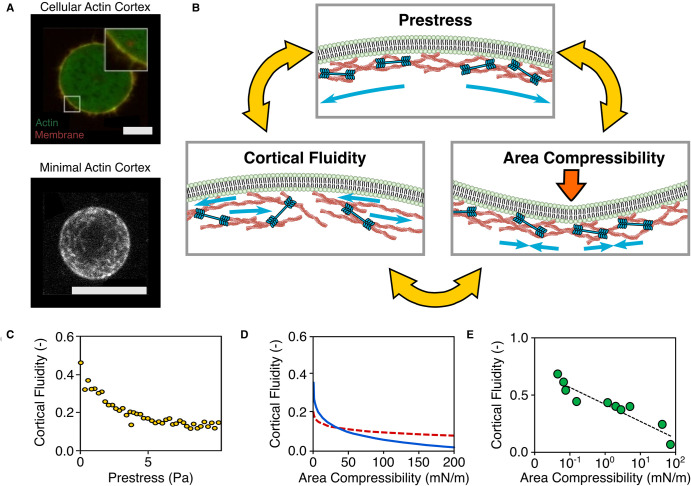
Mechanisms of mechanical reinforcement in actin-membrane assemblies. (**a**) The actin cortex forms a 200 nm submembraneous network of actin, shown here in a HeLa cell [2] (top) which can be recreated in reconstituted systems [27] (bottom). (**b**) Three variables define the actin-membrane mechanics: cortical fluidity describes the degree of dynamic actin remodelling, cortical stiffness describes the area compressibility of the actin network under applied stress, and prestress describes the internal stress in the actin network exerted by myosin II motors that creates cortical tension. A growing body of research shows that these three variables appear to be intrinsically *interdependent*, a finding supported by studies of (**c**) membrane-free actin networks [28] (**d**) nanoindentation of cell membranes and planar minimal actin cortices, shown in the absence (dashed line) and presence (solid line) of ATP [29] and (**e**) nanoindentation studies of live cells [30]. Scale bars are 10 µm.

Perhaps the most fundamental role of the actin-membrane assembly in cells is to provide mechanical support [25]. Internal stresses in the cell's actin cortex are generated by myosin II motors [3], leading to cortical tension. Because this tension is typically nonuniform [26], tension gradients occur, resulting in changes in shape that drive processes such as migration, division and motility. Reconstituted studies, supported by live cell studies have helped to identify three key variables that regulate the cortex mechanics (Figure 2).

The first of these is the cortical stiffness, typically described by the area compressibility modulus K*_A_*, the ratio between the applied stress and the resulting increase in cortical area. In live cells, uniform changes in stiffness induce cell rounding while local changes in stiffness can induce deformations. The lipid membrane appears to have a low compressibility [31] in isolation but is enhanced in the presence of a reconstituted actin cortex [32,33,34], provided that the cortical thickness and extent of actin-membrane coupling are sufficient [35]. This indicates strongly that the membrane only minimally contributes to cortical stiffness, instead acting as a substrate for the stiffer actin cortex. Live cell studies suggest that cortex stiffness can be modulated by the microscopic structure and composition of the constituent actin network [26].

Interestingly, the stiffness of actin cortices in GUVs is typically an order of magnitude lower than that of live cells [32] suggesting that reconstituted systems may be missing some key structural component. One possible explanation for this discrepancy is that the use of physiological linkers such as ezrin have only recently begun to be adopted, therefore, previous work could reflect unrealistic actin-membrane binding strengths and lifetimes. Recent microrheology studies of planar actin cortices have indicated that stiffness is proportional to the surface density of ezrin binding sites, which would support this hypothesis [36].

As well as possessing a stiffness, the cell cortex is a viscoelastic material, whose mechanical properties exhibit a dependence on time and frequency. Extensive studies of live cells using microrheology have established that the cortical stiffness follows a power law dependency [37]. This is a common feature of soft structured materials and indicates a disordered and metastable material. Mechanistically, the power law exponent *β* describes the cortical fluidity, with a high *β* indicating cortical remodelling and a low fluidity indicating a ‘frozen' [38] state, with minimal cortical rearrangement. This power law model provides an attractive and accurate description of live cell viscoelasticity. Live cells typically have reported values of *β* between 0.2 and 0.4. This value decreases when the cell is chemically fixated [30] and increases [39] with mechanical or chemical disruption [30]. One possible mechanism for this increase in *β* is myosin activity which has been shown to modify viscoelasticity in studies of solutions of actin filaments. The activity of myosin led to an increase in fluidity due to the contribution of myosin in sliding actin filaments past each other [40]. In cross-linked reconstituted actin networks, the value of *β* is also dependent on other actin-binding proteins. Rheological experiments on networks cross-linked with *α*-actinin show that *β* increases below a characteristic frequency *ω*_0_ which is a measure of *α*-actinin's unbinding rate [41,28]. Similar experiments on membrane-bound actin networks linked with ezrin show that the unbinding rate of ezrin is an order of magnitude lower than that of *α*-actinin [36]. This suggests that the presence of ezrin increases the attachment timescale of the actin network to the membrane, which, in live cells, may aid the cortex in regulating self-organization and contractility. While these unbinding rates are in approximate agreement with live cell studies [42], increases in *β* at low frequencies have yet to be observed in rheological studies of living cells.

As well as inducing actin network fluidization, myosin II motors exert a measurable internal stress in strongly cross-linked reconstituted actin networks. This stress, typically referred to as the prestress, induces strain stiffening of individual actin filaments as they are pulled in the direction of strain [43]. It is likely that, in live cells, this stiffening mechanism is advantageous in maintaining shape stability [44]. Live cell studies also indicate that the influence of prestress on cell shape can be tuned by the cortex's architectural parameters including cross-linking [45], mesh size, network branching [46], cortical thickness [47] and membrane anchoring.

There is an emerging picture that these three mechanical variables of the cortex; area compressibility, fluidity and prestress are interrelated. Simply from a qualitative perspective, prestressed actin cortices invariably have a lower fluidity and higher stiffness, and stiffer cortices have lower fluidity, suggesting an intrinsic relationship between these three variables. A quantitative basis for this relation has recently been confirmed in both nanoindentation studies of reconstituted actin networks [28] and in reconstituted actin cortices [29]. Significantly, this interrelation of mechanical variables has also been observed in live cells [30] providing compelling evidence that all three variables are fundamental to the actin cortex, are intrinsically interrelated and are presumably tuned in cells in an interdependent manner through changes in actin network architecture.

## Shape deformations

Cells change their shape as a result of a complex interplay between their plasma membranes and the underlying dynamic actin assemblies. Actin monomers polymerize into filaments and depolymerize from the opposite filament end, thus giving rise to cell polarity. These dynamic filaments further cross-link into various actin assemblies. Bound to a membrane, an actin network polymerizes by inserting new monomers at the tip of the filament facing the membrane [48].

This results in an outward pressure onto the membrane and in a retrograde actin flow away from it. In the following, we review studies on minimal actin systems that have provided novel insights into how these processes can shape the cell surface.

**Figure 3.**
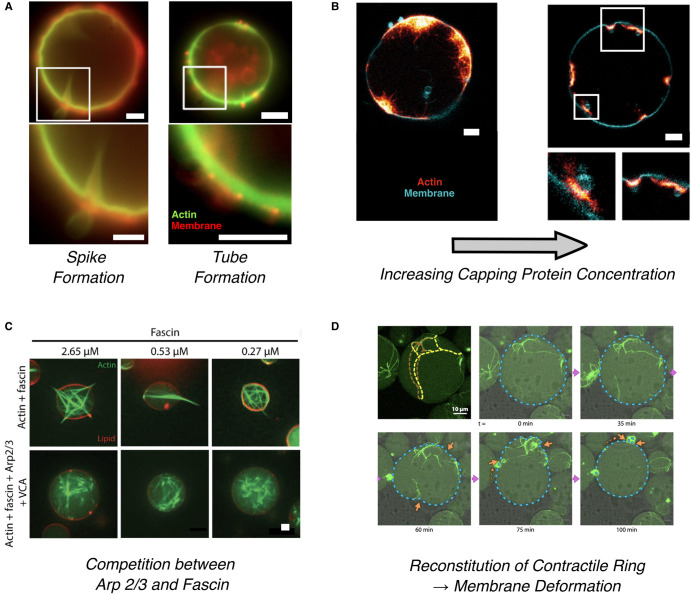
Examples of key cell shape change phenomena that have been recreated in recent work. (**a**) A dynamically polymerizing actin network, branched with arp2/3, bound to the outside of a GUV was shown to be sufficient to induce both inward ‘spike'-like protrusions and outward membrane deformation in the form of tube formation [24]. (**b**) Inward and outward deformation was also created through control of reaction kinetics, using capping proteins, in arp2/3 branched networks bound to the inner leaflet of a GUV. The nature of deformation depended solely on the capping protein concentration, with protrusions occurring at low capping protein concentrations, transitioning to invagination-like deformations at higher concentrations [49]. (**c**) Inner-membrane-bound actin networks with fascin and arp2/3 adopt bundled and dendritic morphologies whose ability to form protrusions is a direct consequence of the relative concentrations of the two binding proteins [50]. (**d**) The formation of contractile actomyosin ring-like structures was achieved through a combination of fascin-induced bundling and myosin-induced contraction in inner-leaflet-bound actin networks [21]. Here, yellow lines indicate the approximate fascin bundle position. All scale bars are 5 µm unless otherwise stated.

Using branched actin networks assembled on the outside of a GUV in the presence of arp2/3 complexes, Simon et al. [24] showed that the friction force arising from the retrograde actin flow is sufficient to pull thin endocytic-like tubes from the membrane (Figure 3a). Coexisting with the tubes, dendritic filopodia-type spikes facing the opposite direction can also form, driven by velocity gradients in the retrograde actin [24]. In a separate study, Dürre et al. demonstrated that controlling the length of the filaments using capping proteins has a similar effect on the type of protrusions (Figure 3b). Low concentrations of capping proteins favour the homogeneous growth of branched networks on the inner side of GUVs, which pushes the membrane into outward protrusions, while high capping protein concentrations induce invagination-like deformations [49]. When the branching arp2/3 complex is replaced by the shorter cross-linking protein fascin, the growing actin filaments adopt a parallel orientation into bundles that deform the membrane into cylindrical fillopodia-like protrusions [5,6]. In addition, model systems have been instrumental in revealing the unappreciated but equally important role of membrane mechanics on actin organization. For example, driven only by the confining effect of the membrane, dendritic actin networks may reorganize into bundles, even in the absence of fascin [23,50]. High membrane stiffness may entirely suppress the formation of filopodia-like protrusions and result instead in the assembly of actin rings on the inner side of vesicles [5] (Figure 3c). Work with water-in-oil droplets confirms that the ring formation is a direct consequence of the spherical confinement of the actin network [51]. More recently it has been shown that enhanced actin-membrane anchoring can also favour the formation of actin rings and their coalescence into a single ring [21] (Figure 3d).

In addition to polymerization/depolymerization, actin networks remodel under the action of myosin II motors, which govern the contraction and disassembly of cellular protrusions, or the formation of contractile actin rings during cell division. In 2D, myosin-induced contractions of actin networks bound to supported planar membranes result in buckling and severing of individual actin filaments [8,19], the latter likely contributing to the mechanisms of actin fluidization observed in cells [52]. If instead of strong actin-membrane anchors, transient ezrin-based linkers are used, the whole actin network contracts and reorganizes into distinct bundles and asters [53]. Combining both actomyosin contractility with actin polymerization in model systems results in a dynamic steady state of constant actin turnover [54], which resembles actin turnover in several cellular contexts.

Actomyosin networks linked to vesicles induce 3D actin and membrane reorganization, although reproducing actual cell shapes has turned out to be more difficult. Cortices contracting on the outer surface of GUVs can either crush the GUV, in the case of dense cortex-membrane linkage, or rupture and peel from the GUV, in the case of sparse linkage [27]. When reconstituted inside GUVs in the presence of the membrane anchor anillin, acto-mysoin cortex contractions result in membrane blebbing [55]. Contrary to cells, however, these blebs remain stable due to the lack of actin polymerization and annealing. Myosin can also lead to the contraction of actin rings. Vesicle division is yet to be achieved, however, because the rings slide on the membrane and collapse into a single condensate, suggesting that additional protein machinery for stabilization may be needed.

## Future outlook

The overarching message from reconstituted studies of actin-membrane assemblies is that such systems provide an excellent adaptable platform for mimicking live cell behaviour in a controllable manner. A major milestone has been the development of physiological membrane anchors between the actin mesh and the membrane, in the form of ezrin-mediated binding. It is increasingly being realized that the extent and lifetime of anchoring is an integral factor in mimicking the native mechanics of the live cell cortex. We note that most reconstituted studies do not utilize ezrin anchoring, and that recent work has shown that this anchoring may affect the architecture of actin networks and may even promote force generation [18]. As this review moreover shows, certain cell deformations can be achieved using various sets of components *in vitro*. It remains to be further clarified whether and how certain processes in cells prevail over others or whether biological systems maintain on purpose such broad parameter space.

A prominent future challenge in synthetic biology remains the reproduction of complex cellular phenomena such as cell division and motility [56]. In the case of motility, for example, it would be necessary to induce the lamellipodia and filopodia at the leading cell edge, the attachment of these protrusions to a substrate and the subsequent triggering of actomyosin contraction, that contracts the rear end of the cell and also contributes to the actin turnover [57,58]. To spatially and temporally co-ordinate the necessary processes many biophysical tools are likely to be useful in this regard including the ability to trigger myosin activity through the light-induced inactivation of inhibitor blebbistatin [59], chemically trigger changes to membrane composition through reagents such as cyclodextrin [60], or use PIP2 producing kinases to dynamically alter anchoring [61].

Also unexplored, is the mechanical contributions of cytoskeletal filaments other than actin. Microtubules, which drive cell polarity in living cells, are known to dynamically co-ordinate with actin filaments to control cell polarity and may also modulate the cortical stiffness [62]. Septin filaments [63], which act as a scaffold for actomyosin contraction [64], have recently been successfully reconstituted in GUVs [65]. Although the septins did not appear to alter the cortical stiffness, they were observed to induce deformations in GUVs and, like ezrin, have been shown to bind to PIP2 lipids [66], suggesting that many of the design strategies of actin networks could be generalized to septins. Intermediate filaments, which act as scaffolds and provide resilience to large cellular deformations, are also increasingly recognized as interacting with the plasma membrane [67] and intriguingly may play a role in regulating cortical thickness [68].

## Summary

- Reconstituted membrane-actin assemblies are an adaptable platform for mimicking live cell mechanics and recreating shape change phenomena such as membrane protrusions and contraction.
- Key mechanical properties comprise the stiffness, fluidity and prestress of actin cortex, each of which is interdependent and also regulated by actin architecture.
- Many cellular deformations have been successfully reproduced. However, understanding their dominant mechanisms in the existing wide parameter space has yet to be achieved. This includes considerations of other cytoskeletal proteins, particularly actin-binding proteins.
- The reconstitution of more complex cellular processes such as division and motility will require careful spatial and temporal control and coordination of simpler processes that have already been reconstituted.

## Open Access

Open access for this article was enabled by the participation of Durham University in an all-inclusive *Read & Publish* agreement with Portland Press and the Biochemical Society under a transformative agreement with JISC.
